# Environmental Sustainable Cement Mortars Based on Polyethylene Terephthalate from Recycling Operations

**DOI:** 10.3390/ma16052111

**Published:** 2023-03-06

**Authors:** Francesco Todaro, Andrea Petrella, Giusy Santomasi, Sabino De Gisi, Michele Notarnicola

**Affiliations:** Department of Civil, Environmental, Land, Building Engineering and Chemistry (DICATECh), Polytechnic University of Bari, Via E. Orabona n. 4, 70125 Bari, Italy

**Keywords:** cement mortars, polyethylene terephthalate, rheology, thermal insulation, mechanical strength, microstructural properties

## Abstract

The building and construction industry is a key sector behind the ecological transition in that it is one of the main responsible factors in the consumption of natural resources. Thus, in line with circular economy, the use of waste aggregates in mortars is a possible solution to increase the sustainability of cement materials. In the present paper, polyethylene terephthalate (PET) from bottle scraps (without chemical pretreatment) was used as aggregate in cement mortars to replace conventional sand aggregate (20%, 50% and 80% by weight). The fresh and hardened properties of the innovative mixtures proposed were evaluated through a multiscale physical-mechanical investigation. The main results of this study show the feasibility of the reuse of PET waste aggregates as substitutes for natural aggregates in mortars. The mixtures with bare PET resulted in less fluid than the specimens with sand; this was ascribed to the higher volume of the recycled aggregates with respect to sand. Moreover, PET mortars showed a high tensile strength and energy absorption capacity (with Rf = 1.9 ÷ 3.3 MPa, Rc = 6 ÷ 13 MPa); instead, sand samples were characterized by a brittle rupture. The lightweight specimens showed a thermal insulation increase ranging 65–84% with respect to the reference; the best results were obtained with 800 g of PET aggregate, characterized by a decrease in conductivity of approximately 86% concerning the control. The properties of these environmentally sustainable composite materials may be suitable for non-structural insulating artifacts.

## 1. Introduction

Industrial waste disposal is certainly a source of economic, environmental, social and health issues. Accordingly, the reuse and recycling operations represent a new development opportunity to manage a large quantity of solid waste [1,2,3,4,5,6].

From the 1950s to today, the production of plastic materials has grown exponentially and is destined to grow dramatically in the coming decades [7,8].

There are many types of plastics, used in the most disparate sectors of human activities, such as polyethylene, polypropylene, polyvinyl chloride, polystyrene, polyamides and polyethylene terephthalate.

The polyethylene terephthalate (PET) is a thermoplastic polymer belonging to polyesters and is one of the most widely used plastics in the package industry whose production has been increasing continuously since the late 1970s. This material has found strong applications in place of glass in the production of bottles due to its lightness and ease of handling and storing. PET containers are used not only for mineral water, but also for fruit juices, soft drinks, milk, etc., since they have numerous advantages in addition to lightness, such as cheapness, chemical inertia, high pressure tolerance and impermeability to gases.

PET is a resistant material, and with an estimated average life of around 1000 years, it can be considered non-biodegradable. For this reason, the disposal of this product is a problem to be solved since the dispersion of plastics in the environment cause problems to the habitat of fauna and flora and therefore to the humans through the food chain [9,10].

The containment of the environmental impact of plastic materials is therefore achieved through a dual approach as reduction of packaging by producers and correct disposal by consumers.

For this reason, it is very important the plastic disposal goes through separate collection in order to transform this type of waste into a resource.

There are different methods for disposing such materials: incineration, landfilling and recycling. Incineration can produce enough heat and steam to generate electricity for the local grid and it represents an easy and cheap solution, but with a harsh impact on air, water and soil quality [11,12]. Moreover, since plastic decomposes much more slowly than any other waste, landfilling cannot be considered an efficient method of disposal because hundreds of years are needed for a natural decomposition [13].

As a matter of fact, waste recycling is the best way for sustainable management of plastics because of environmental compatibility and economic benefits [14,15].

An economical way of PET recycling is the incorporation in cement conglomerates as granulate after mechanical grinding [16,17,18]. Some researchers investigated the main properties of mortars with PET. In the study of Yilmaz [19] waste PET granules were used at a 5%, 10% and 20% replacement of the sand. His study indicated some drawbacks of the use of waste PET aggregates, such as the decrease in compressive strength and fire resistance, but also some benefits, such as a reduction in the density and water absorption and high frost resistance of the cement mortar. Results were confirmed in other later studies [20,21,22].

As a result, PET used as aggregate in mortars and concrete formulation can increase the sustainability of this waste with many positive effects, such as decrease in the usage of natural resources, waste consumption, environmental protection and energy saving [23,24]. In most cases, however, chemical treatments were investigated to improve the surface quality of plastic aggregates, with cost and environmental impacts inadequate [25,26,27].

In this research, cheap, environmentally friendly and lightweight cement mortars were prepared with recycled polyethylene terephthalate (PET) as an aggregate deriving from bottle scraps. Moreover, no treatments of this material and of the mixture occurred. Specifically, different types of conglomerates were studied in fresh (rheology) and hardened state (mechanical, thermal, and microstructural properties) in order to show the peculiar characteristics of each composite. The specimens were prepared with partial and total replacement of the conventional sand aggregate with PET grains in the range of 1–2 mm. A comparison among mortars with bare PET, bare sand and mixture of aggregates was obtained to understand the potential applications of these materials.

## 2. Materials and Methods

The preparation of the cement mortars was obtained with CEM II A-LL 42.5 R (Buzzi Unicem, Barletta, Italy) cement [28]. Polyethylene terephthalate (PET) from bottle scraps (Maltek Industrie Srl, Terlizzi, Italy) was crushed (with the cutting mill SM 300, Retsch GmbH) and used after sieving (with electromagnetic sieve shaker IRIS FTL-0200, FILTRA Sl, Barcelona, Spain) with grain sizes in the range of 1–2 mm, which partially and totally substituted sand aggregate. Normalized sand (~1700 g/dm^3^, 0.08–2 mm, Societè Nouvelle du Littoral, Leucate, France) was used in the mortars with grains in the range of 0.5–1 mm after sieving.

**Figure 1:**
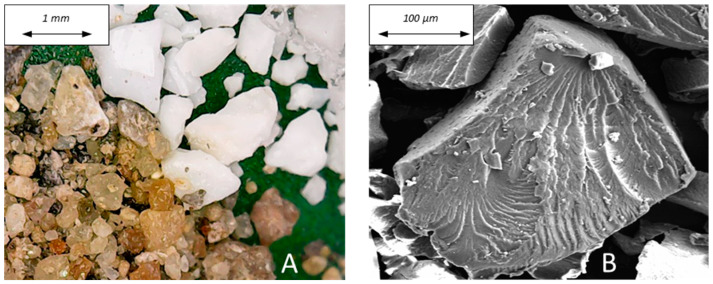
(**A**) Sand (left) and PET (right) grains and (**B**) SEM image of a PET grain.

Figure 1A shows the inorganic (sand) and the organic (recycled PET) grains which were used as aggregates for the mortars’ preparation. The SEM image of a PET grain is reported in Figure 1B.

Table 1 shows the samples, prepared with 225 g of water and 450 g of cement [29], with the same water to cement ratio of the reference sample (normalized mortar) which was prepared with normalized sand (1350 g).

A flow-test allowed for determining the consistency of the samples in the fresh state [30]. The percentage increase in the diameter (flow) was calculated with Equation (1):

flow = [(D_2_ − D_1_)/D_1_] × 100 (1)

where D_2_ is average spread diameter and D_1_ is the original base diameter.

After 28 days of curing, specimens were mechanically characterized with a compression and flexural testing machines (MATEST, Milan, Italy). Specifically, 40 × 40 × 160 mm prisms were prepared, and cured for 28 days. Successively, flexural and compressive tests were carried out, respectively, with 50 ± 10 N/s and 2400 ± 200 N/s loading rates [29].

The hardened specimens were also thermally characterized with a portable system for measurement of heat transfer properties of materials (ISOMET, Applied Precision Ltd., Bratislava, Slovakia). Specifically, φ = 100 mm; h = 50 mm cylinders were prepared and cured for 28 days. A thermal stress was induced on the sample from a flat source which was placed on the surface. This allowed for obtaining an estimation of the thermal conductivity (λ) of the mortars [31].

The composites were also microstructurally characterized with a FESEM-EDX Carl Zeiss Sigma 300 VP electron microscope (Carl Zeiss Microscopy GmbH, Jena, Germany), after application of the materials onto aluminium stubs and gold sputtering. The aggregate distribution of the mortars was observed with a Premier series dyno-lyte portable microscope.

## 3. Results and Discussion

### 3.1. Preliminary Remarks

Table 1 shows that the PET mortars (PET1, PET2, PET3, samples 7, 8 and 9, respectively) were lighter and more porous than the sand samples (SAND1, SAND2, SAND3, samples 1, 2 and 3), and also in comparison with the reference (control), which showed the highest density among the tested specimens [32,33,34,35]. Among the lightweight mortars, the PET1 composite was the most porous and the lightest because of the largest quantity of organic aggregate (800 g) added into the mixture. The PET2 and PET3 mortars were prepared with 500 g and 250 g, respectively. Samples with an aggregate mixture (SP20, SP50, SP80, samples 4, 5 and 6) showed a decrease in specific mass and increase in porosity with the increase in the lightweight aggregate (PET) and the SP50 sample showed intermediate values of specific mass and porosity, constant the final aggregate dosage, 500 g, in the mixture (PET2 and SAND2).

### 3.2. Rheology

**Figure 2:**
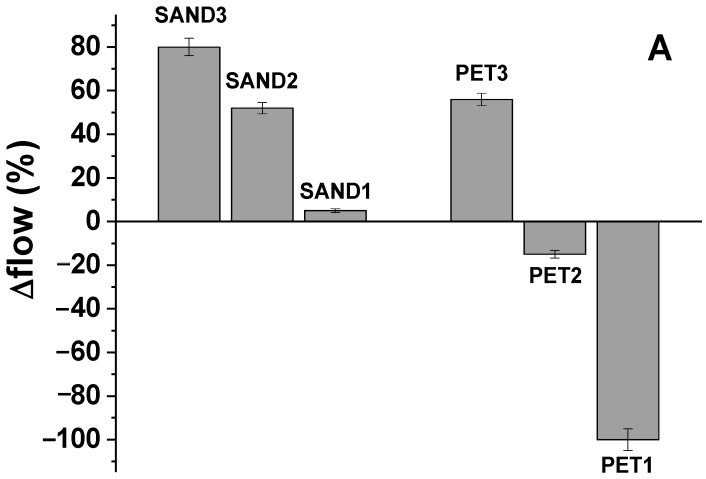

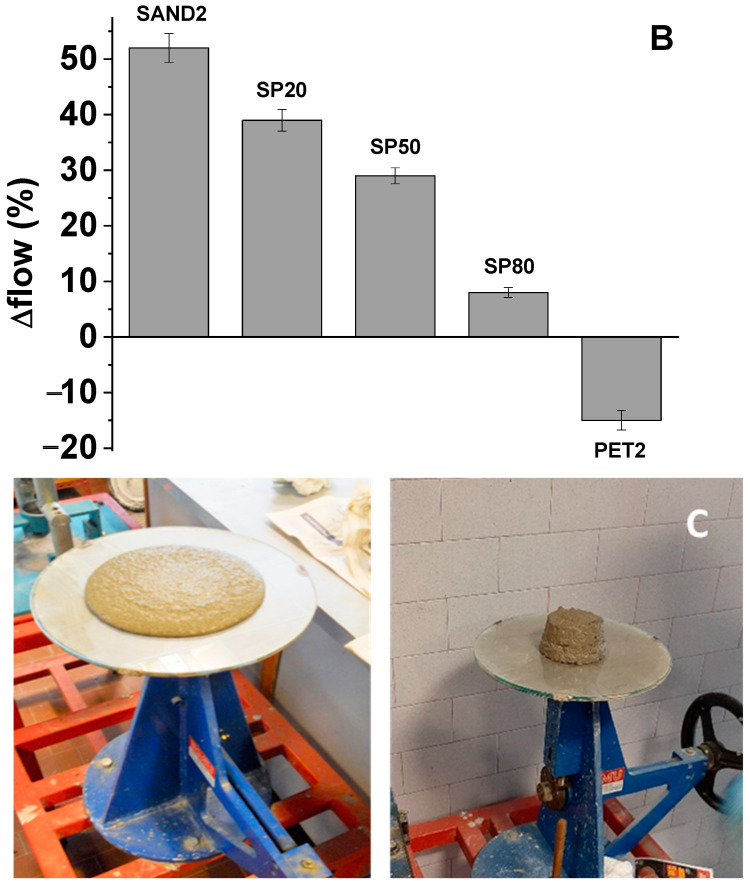
(**A**) Flows of the SAND and PET composites respective to the normalized mortar, (**B**) flows of the SP, SAND2 and PET2 composites respective to the normalized mortar, (**C**) flow tests of the SAND1 (left) and of the PET1 samples (right).

The flow-test allowed for determining the consistency of the fresh specimens (Figure 2) and the values were compared with the result obtained by the control (Equation (2)).

Δflow = [(flow_specimen_ − flow_normalized mortar_)/flow_normalized mortar_] × 100 (2)

SAND1 (Figure 2A) showed similar flow of the control (+5%, plastic behaviour), whereas with lower sand dosage, the samples resulted in becoming increasingly more fluid (SAND2 (+52%) and SAND3 (+80%), respectively, with 500 g and 250 g of sand). The mixtures with bare PET resulted in less fluid than the sand samples. Specifically, the PET3 mixture (with 250 g of aggregate) showed an increase in fluidity in the range of +56% with respect to the control, but a decrease with respect to SAND3 (with 250 g of aggregate). The PET2 mixture resulted in a similar workability of the control (−15%, plastic behaviour) but was more dried than SAND2, which was characterized by the same aggregate dosage (500 g).

Finally, the PET1 mixture was extremely dried, with rheological features totally different with respect to SAND1, which was characterized by the same aggregate quantity (800 g).

These results can be associated with the absorption of water promoted by PET aggregate [36,37], which contributed to a decrease in the fluidity of the specimens sequestering the free water; this effect is more evident with the increase in PET dosage.

Specimens with a mixture of aggregates (SP samples) resulted in a decrease in fluidity with the increase in PET (Figure 2B), and SP80 resulted in a similar workability of the normalized mortar (+8%), while SP50 showed an intermediate value (+29%) with respect to bare aggregate samples, SAND2 (+52%) and PET2 (−15%). Figure 2C shows a picture of the flow tests of the SAND1 (left) and of the PET1 samples, respectively, evidencing the different rheological behaviour of composites characterized by the same dosage but different types of aggregates.

The workability of fresh mortars determines the properties of hardened materials; indeed, the lack of workability prevents the molding of specimens from taking place efficiently, compromising mechanical resistance [36]. Similar results were shown by Albano et al. [37], where a PET content in 20% decreased both the cement mortars’ fluidity and mechanical strengths.

### 3.3. Mechanical Strengths

**Figure 3:**
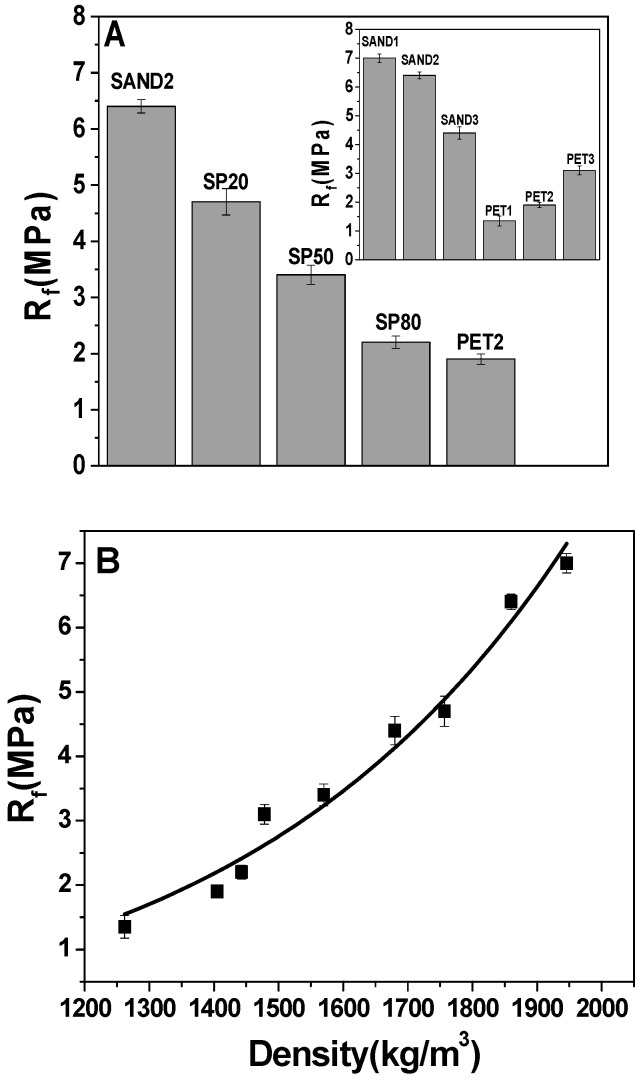

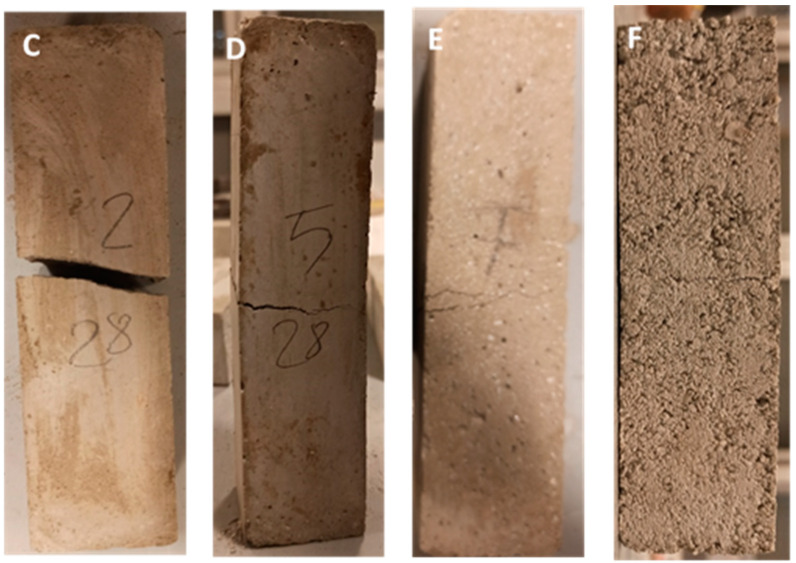
(**A**) Flexural strengths of SAND2, SP and PET2 samples (with 500 g of aggregate). In the inset: flexural strengths of bare SAND and PET specimens. (**B**) Exponential increase in the flexural strengths with density. Flexural cracks in (**C**) SAND1 sample, (**D**) SP50 sample, (**E**) PET2 sample, (**F**) PET1 sample.

Figure 3 reports the flexural strengths of the samples. The values of the SAND composites increased with the increase in the aggregate dosage (~4.5–7.0 MPa), whereas the PET composites showed lower strengths (~1.3–3.0 MPa) above all in comparison with the control (~8.6 MPa), because all the sand samples were characterized by a more resistant aggregate, thus showing higher densities [38]. These results can be attributed to the negative effect of PET smooth surface texture on the bond strength between cement and aggregates [39].

Moreover, the lightweight specimens showed an increase in the strengths with the decrease in PET. Accordingly, PET3 resulted in being the most resistant (3.1 MPa), and with values more than double than PET1 (1.35 MPa) because of having the highest density and the lowest porosity. SP samples’ flexural resistances were between the SAND2 and PET2 values (6.4 MPa and 1.9 MPa, respectively), and decreased with the organic aggregate increase due to the specific mass decrease in the composites, together with the increase in the porosity. The flexural strengths showed an exponential increase with the density increase in the composites. Moreover, the sand samples showed a brittle rupture (Figure 3C) which became semi-brittle in the SP50 (Figure 3D) and PET3 conglomerates. Instead, for PET2 and especially in PET1 mortars was observed (Figure 3E,F) a ductile fracture without specimen collapse (where the plastic aggregates prevent the propagation of cracks), associated to the high tensile strength and energy absorption capacity of PET [40,41].

As shown in Figure 3F, the PET particles can make some interlock between the two fractured surfaces because of the special shape of PET particles and their flexibility, which prevented the complete beam failure.

The PET composites’ results are consistent with other research that has identified for mortars with a max 50% of recycled PET, a flexural strength of between 1 and 4.5 MPa [20].

**Figure 4:**
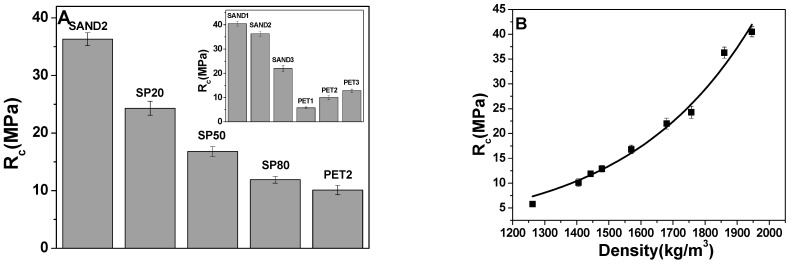
(**A**) Compressive strengths of SAND2, SP and PET2 samples (with 500 g of aggregate). In the inset: compressive strengths of bare SAND and PET specimens. (**B**) compressive strengths vs. sample density.

Figure 4 reports the compressive strengths of the samples. With the increase in the sand dosage, the resistances of the SAND composites tend to increase with values that in the case of the SAND1 sample were similar to the normalized mortar (40.5 Mpa vs. 47 Mpa). The PET aggregate addition was determined to decrease the compressive strengths, which was evident in the case of the SP80 composite (~12 Mpa). Bare PET specimens showed the lowest strengths, except in the case of the PET3 sample (~13 Mpa), due to the lowest quantity of aggregate added (250 g) with respect to the other similar composites. PET2 and PET3 resulted in being more resistant than PET1 (~10 Mpa and ~13 Mpa vs. ~6 Mpa), due to the higher porosity of the latter mortar associated to the higher dosage of PET (800 g) which is evidenced in Figure 3F. Additionally, in this case, the compressive strengths showed an exponential increase in the compressive strengths with the density increase and discrete cracks with no collapse in the PET samples.

This trend can be attributed to the decrease in adhesive strength between the surface of the plastic waste and the cement paste [16,21,27,42]. An increase in the roughness of the aggregates’ surface may increase the compressive strengths [26,27].

**Figure 5:**
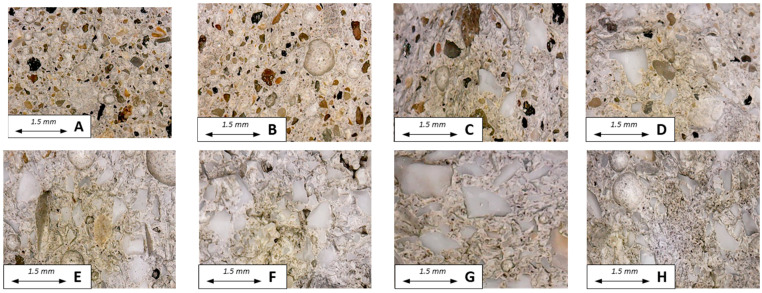
Sections of (**A**) SAND1, (**B**) SAND2, (**C**) SP20, (**D**) SP50, (**E**) SP80, (**F**) PET1, (**G**) PET2, (**H**) PET3.

The mortars’ sections are observed in Figure 5, where the different aggregates compositions were showed. Specifically, Figure 5A,B show the decrease in the sand dosage from SAND1 to SAND3 samples, whereas Figure 5C–E show the increase in the PET concentration from SP20 to SP80 samples. Finally, it can be observed that for bare PET mortars, an increase in the PET dosage determined an increase in the porosity, which is clearly evidenced in the PET1 composite (Figure 5F–H).

The values obtained are compatible with other similar studies [20,42].

### 3.4. Thermal Conductivity

**Figure 6:**
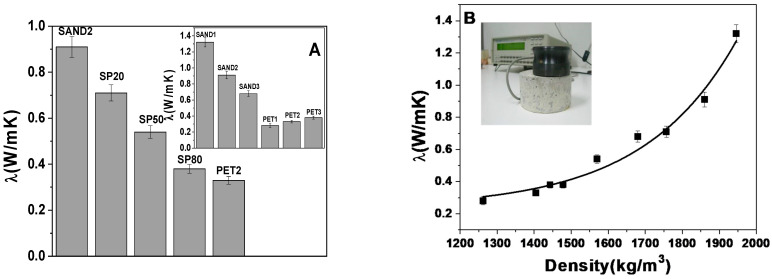
(**A**) Thermal conductivities of SAND2, SP and PET2 samples (with 500 g of aggregate). In the inset: thermal conductivities of bare SAND and PET specimens. (**B**) thermal conductivities vs. sample density.

Figure 6 reports the thermal conductivity of the samples. Sand-based specimens resulted in being more insulating than the normalized mortar [29,30]. In fact, these conglomerates showed values ranging 0.7–1.3 W/mK with respect to ~2 W/mK of the control. An increase in the values was observed with the increase in the conventional aggregate dosage which is responsible of the increase in density of these types of conglomerates. The addition of the recycled plastic material determined a decrease in the thermal conductivity of the SP samples associated both to a density decrease and porosity increase. Specifically, values ranging 0.3–0.7 W/mK were observed for these composites, with a thermal insulation increase in 65–84% with respect to the reference.

The best results were obtained with PET conglomerate, characterized by a decrease in conductivity of approximately 86% with respect to the control. As already showed in Figure 5G, the relevant porosity of this sample can explain this result. Among bare PET specimens, PET3 was the least insulating (~0.39 W/mK), due to the lowest dosage of aggregate, which induced a specific mass increase and a porosity decrease.

An exponential increase in the conductivity with the density increase in the mixtures was observed [32,33] (Figure 6B).

### 3.5. Microstructural Properties

**Figure 7:**
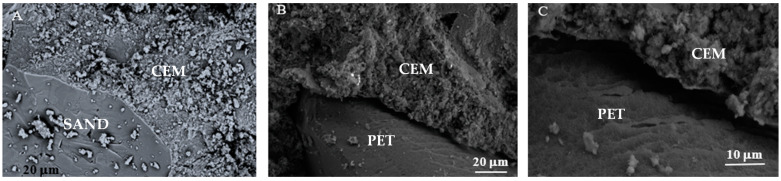
Scanning electron microscope detections of: (**A**) sand/cement interface in the SAND2 composite, (**B**) PET/cement interface in the PET2 composite, (**C**) magnification of the PET/cement interface from (**B**).

The low mechanical strengths, together with the high thermal insulation of bare PET samples, can be explained with scanning electron microscope detections (Figure 7). It can be observed that the sand adhesion to the cement matrix (Figure 7A), on the contrary to what was observed with PET grains which, due to hydrophobic features, did not adhere in the same manner, generating voids at the interface [26,43,44,45] (Figure 7B). The size of these voids tends to be wider with the increase in the PET dosage. For example, in the case of the PET2 composite, voids in the range of 5µm can be observed (Figure 7C). Accordingly, the simultaneous presence of the lightweight recycled materials in the mixtures, combined with the low adhesion to the cement paste, determined a great decrease in the density of the composites with an increase in the porosity (Table 1), which determined the low strengths and the high thermal insulation [32,33,34,35,36].

**Figure 8:**
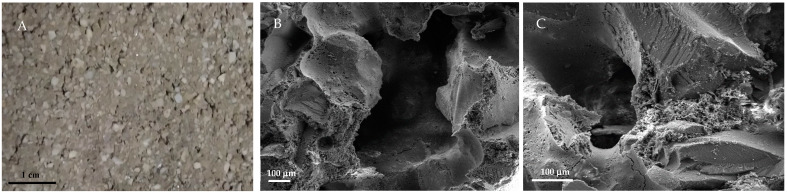
(**A**) Image of the PET1 (800 g of PET) sample surface, (**B**,**C**) SEM detections of the PET1 sample.

The further increase in the organic aggregate generated a strong increase in the porosity, as in the case of the PET1 sample (Figure 8A). Specifically, SEM detections of Figure 8B,C evidenced pores in the matrix ranging 500–800 µm.

For all these reasons, the simultaneous presence of the conventional and recycled aggregates in the SP mortars determined intermediate values of mechanical resistances and thermal conductivity.

As a final remark, the PET2 and PET3 samples, characterized by 500 g and 250 g of recycled aggregate, respectively, are a good compromise among rheological, mechanical and thermal properties. PET3 is more resistant and more fluid than PET2, characterized by a lower thermal conductivity and with a workability very similar to the normalized mortar.

## 4. Conclusions

Polyethylene terephthalate (PET) from bottle scraps was used as aggregate in lightweight cement mortars which were characterized with rheological, mechanical, thermal and microstructural tests. The following conclusions can be drawn based on experimental results compared with studies reported in the literature:

− the mixtures with bare PET resulted in less fluid than the sand samples (SAND), which is ascribed to the higher volume of the recycled materials respective to sand.
− SP samples resulted in a decrease in fluidity with the increase in PET dosage and SP80 resulted in a similar workability of the normalized mortar (+8%), while SP50 showed an intermediate value (+29%) with respect to the samples with bare aggregate.
− The PET composites showed lower strengths (Rf = 1.9–3.3 MPa, Rc = 6–13 MPa) than the SAND samples and the reference, with results increasing with the decrease in the dosage of the organic aggregate. SP conglomerates showed intermediate values (Rf = 2.2–4.7 Mpa, Rc = 11.9–24.3 Mpa)
− The sand samples showed a brittle rupture which became semi-brittle in the SP50 and PET3 conglomerates and was not observed in PET2 and especially PET1 mortars. Both were characterized by discrete cracks with no collapse, associated to the high tensile strength and energy absorption capacity of PET.
− The addition of PET determined a decrease in the thermal conductivity (0.3–0.7 W/mK), associated to the density decrease and porosity increase. The thermal insulation enhancement was ~65–84% compared to the reference (~2 W/mK). The best results were obtained with PET1 conglomerate (800 g PET), the most porous. The thermal and mechanical results can be explained through microstructural detections which revealed the low adhesion of PET to the cement mixture; accordingly, the simultaneous presence of the lightweight recycled materials combined with the low adhesion to the cement paste resulted in a great decrease in the specific mass of the composites and the increase in porosity.
− The SP and PET specimens are effectively environmentally sustainable conglomerates due to the preparation with products derived from bottle scraps and due to the lightness, which is fundamental for thermal insulating materials in non-structural structures.

An example of a possible application for lightweight cement mortars with PET aggregates is in civil engineering applications, such as elements with efficient thermal insulation or non-structural concrete panels. For this purpose, guidelines for the use of waste plastic aggregate in cement mortars are essential for reliable design and construction. Finally, more research is required to grow confidence in using recycled aggregates (e.g., the long-term properties of concrete with PET aggregates still need to be appropriately investigated).

## Figures and Tables

**Table 1:** Composition, density *ρ* and porosity of the cement mortars.

Sample	Type	Cement (g)	Water (cm^3^)	PET (g)	Sand (g)	Density (kg/m^3^)	Porosity (%)
Control	normalized mortar	450	225	0	1350	2060	22
1	SAND1	450	225	0	800	1946	23
2	SAND2	450	225	0	500	1860	23
3	SAND3	450	225	0	250	1680	24
4	SP20	450	225	100	400	1757	28
5	SP50	450	225	250	250	1570	31
6	SP80	450	225	400	100	1443	35
7	PET1	450	225	800	0	1262	41
8	PET2	450	225	500	0	1405	35
9	PET3	450	225	250	0	1478	31

## Data Availability

The data presented in this study are available on request from the corresponding author.

## References

[1] Salmenperä H., Pitkänen K., Kautto P., Saikku L. (2021). Critical Factors for Enhancing the Circular Economy in Waste Management. J. Clean. Prod..

[2] Prajapati P., Varjani S., Singhania R.R., Patel A.K., Awasthi M.K., Sindhu R., Zhang Z., Binod P., Awasthi S.K., Chaturvedi P. (2021). Critical Review on Technological Advancements for Effective Waste Management of Municipal Solid Waste—Updates and Way Forward. Environ. Technol. Innov..

[3] Nanda S., Berruti F. (2021). Municipal Solid Waste Management and Landfilling Technologies: A Review. Environ. Chem. Lett..

[4] Asefi H., Lim S. (2017). A Novel Multi-Dimensional Modeling Approach to Integrated Municipal Solid Waste Management. J. Clean. Prod..

[5] Rizzi V., D’Agostino F., Gubitosa J., Fini P., Petrella A., Agostiano A., Semeraro P., Cosma P. (2017). An Alternative Use of Olive Pomace as a Wide-Ranging Bioremediation Strategy to Adsorb and Recover Disperse Orange and Disperse Red Industrial Dyes from Wastewater. Separations.

[6] Ranieri E., Tursi A., Giuliano S., Spagnolo V., Ranieri A.C., Petrella A. (2020). Phytoextraction from Chromium-Contaminated Soil Using Moso Bamboo in Mediterranean Conditions. Water Air Soil Pollut..

[7] Zhao X., Korey M., Li K., Copenhaver K., Tekinalp H., Celik S., Kalaitzidou K., Ruan R., Ragauskas A.J., Ozcan S. (2022). Plastic Waste Upcycling toward a Circular Economy. Chem. Eng. J..

[8] Getor R.Y., Mishra N., Ramudhin A. (2020). The Role of Technological Innovation in Plastic Production within a Circular Economy Framework. Resour. Conserv. Recycl..

[9] Raheem A.B., Noor Z.Z., Hassan A., Abd Hamid M.K., Samsudin S.A., Sabeen A.H. (2019). Current Developments in Chemical Recycling of Post-Consumer Polyethylene Terephthalate Wastes for New Materials Production: A Review. J. Clean. Prod..

[10] Law K.L., Narayan R. (2022). Reducing Environmental Plastic Pollution by Designing Polymer Materials for Managed End-of-Life. Nat. Rev. Mater..

[11] Escobar-Arnanz J., Mekni S., Blanco G., Eljarrat E., Barceló D., Ramos L. (2018). Characterization of Organic Aromatic Compounds in Soils Affected by an Uncontrolled Tire Landfill Fire through the Use of Comprehensive Two-Dimensional Gas Chromatography–Time-of-Flight Mass Spectrometry. J. Chromatogr. A.

[12] Artíñano B., Gómez-Moreno F.J., Díaz E., Amato F., Pandolfi M., Alonso-Blanco E., Coz E., García-Alonso S., Becerril-Valle M., Querol X. (2017). Outdoor and Indoor Particle Characterization from a Large and Uncontrolled Combustion of a Tire Landfill. Sci. Total Environ..

[13] van Fan Y., Jiang P., Tan R.R., Aviso K.B., You F., Zhao X., Lee C.T., Klemeš J.J. (2022). Forecasting Plastic Waste Generation and Interventions for Environmental Hazard Mitigation. J. Hazard Mater..

[14] Shamsuyeva M., Endres H.J. (2021). Plastics in the Context of the Circular Economy and Sustainable Plastics Recycling: Comprehensive Review on Research Development, Standardization and Market. Compos. Part C Open Access.

[15] Oberoi I.S., Rajkumar P., Das S. (2021). Disposal and Recycling of Plastics. Mater. Today Proc..

[16] Akçaözoğlu S., Atiş C.D., Akçaözoğlu K. (2010). An investigation on the use of shredded waste PET bottles as aggregate in lightweight concrete. Waste Manag..

[17] Hossain M.B., Bhowmik P., Shaad K.M. (2016). Use of waste plastic aggregation in concrete as a constituent material. Progress. Agric..

[18] Pereira E.L., de Oliveira Junior A.L., Fineza A.G. (2017). Optimization of mechanical properties in concrete reinforced with fibers from solid urban wastes (PET bottles) for the production of ecological concrete. Constr. Build. Mater..

[19] Yılmaz A. (2021). Mechanical and durability properties of cement mortar containing waste pet aggregate and natural zeolite. Ceram. Silikáty.

[20] Campanhão A.F., Marvila M.T., de Azevedo A.R., da Silva T.R., Fediuk R., Vatin N. (2021). Recycled pet sand for cementitious mortar. Materials.

[21] Marvila M., de Matos P., Rodríguez E., Monteiro S.N., de Azevedo A.R. (2022). Recycled aggregate: A viable solution for sustainable concrete production. Materials.

[22] Ahmad F., Jamal A., Mazher K.M., Umer W., Iqbal M. (2022). Performance evaluation of plastic concrete modified with e-waste plastic as a partial replacement of coarse aggregate. Materials.

[23] Mohan H.T., Jayanarayanan K., Mini K.M. (2021). Recent Trends in Utilization of Plastics Waste Composites as Construction Materials. Constr. Build. Mater..

[24] Pablo Ojeda J. (2021). A Meta-Analysis on the Use of Plastic Waste as Fibers and Aggregates in Concrete Composites. Constr. Build. Mater..

[25] Saikia N., De Brito J. (2014). Mechanical properties and abrasion behaviour of concrete containing shredded PET bottle waste as a partial substitution of natural aggregate. Constr. Build. Mater..

[26] Lee Z.H., Paul S.C., Kong S.Y., Susilawati S., Yang X. (2019). Modification of waste aggregate PET for improving the concrete properties. Adv. Civ. Eng..

[27] Almeshal I., Tayeh B.A., Alyousef R., Alabduljabbar H., Mohamed A.M. (2020). Eco-friendly concrete containing recycled plastic as partial replacement for sand. J. Mater. Res. Technol..

[28] Cement Composition, Specifications and Conformity Criteria for Common Cements.

[29] Methods of Testing Cement-Part 1: Determination of Strength.

[30] Determination of Consistency of Cement Mortars Using a Flow Table. https://store.uni.com/uni-7044-1972.

[31] Gustafsson S.E. (1991). Transient Plane Source Techniques for Thermal Conductivity and Thermal Diffusivity Measurements of Solid Materials. Rev. Sci. Instrum..

[32] Petrella A., Petrella M., Boghetich G., Petruzzelli D., Calabrese D., Stefanizzi P., de Napoli D., Guastamacchia M. (2007). Recycled Waste Glass as Aggregate for Lightweight Concrete. Proc. Inst. Civ. Eng. Constr. Mater..

[33] Petrella A., Notarnicola M. (2021). Lightweight Cement Conglomerates Based on End-of-Life Tire Rubber: Effect of the Grain Size, Dosage and Addition of Perlite on the Physical and Mechanical Properties. Materials.

[34] Petrella A., Stefanizzi P., Petrella M., Calabrese D., Boghetich G., Petruzzelli D., Petrella A., Petrella M., Boghetich G., Petruzzelli D. (2009). Thermo-Acoustic Properties of Cement-Waste-Glass Mortars. Proc. Inst. Civ. Eng. Constr. Mater..

[35] Petrella A., Petruzzelli V., Basile T., Petrella M., Boghetich G., Petruzzelli D. (2010). Recycled Porous Glass from Municipal/Industrial Solid Wastes Sorting Operations as a Lead Ion Sorbent from Wastewaters. React. Funct. Polym..

[36] Zhang P., Zheng Y., Wang K., Zhang J. (2018). A review on properties of fresh and hardened geopolymer mortar. Compos. Part B Eng..

[37] Albano C., Camacho N., Hernandez M., Matheus A., Gutierrez A. (2009). Influence of content and particle size of waste pet bottles on concrete behavior at different w/c ratios. Waste Manag..

[38] Petrella A., di Mundo R., Notarnicola M. (2020). Recycled Expanded Polystyrene as Lightweight Aggregate for Environmentally Sustainable Cement Conglomerates. Materials.

[39] Coppola L., Beretta S., Bignozzi M.C., Bolzoni F., Brenna A., Cabrini M., Candamano S., Caputo D., Carsana M., Cioffi R. (2022). The Improvement of Durability of Reinforced Concretes for Sustainable Structures: A Review on Different Approaches. Materials.

[40] Dawood A.O., AL-Khazraji H., Falih R.S. (2021). Physical and Mechanical Properties of Concrete Containing PET Wastes as a Partial Replacement for Fine Aggregates. Case Stud. Constr. Mater..

[41] Asdollah-Tabar M., Heidari-Rarani M., Aliha M.R.M. (2021). The Effect of Recycled PET Bottles on the Fracture Toughness of Polymer Concrete. Compos. Commun..

[42] Islam M.J., Meherier M.S., Islam A.R. (2016). Effects of waste PET as coarse aggregate on the fresh and harden properties of concrete. Constr. Build. Mater..

[43] Petrella A., Petrella M., Boghetich G., Basile T., Petruzzelli V., Petruzzelli D. (2012). Heavy Metals Retention on Recycled Waste Glass from Solid Wastes Sorting Operations: A Comparative Study among Different Metal Species. Ind. Eng. Chem. Res..

[44] Petrella A., Petruzzelli V., Ranieri E., Catalucci V., Petruzzelli D. (2016). Sorption of Pb(II), Cd(II), and Ni(II) From Single- and Multimetal Solutions by Recycled Waste Porous Glass. Chem. Eng. Commun..

[45] Kangavar M.E., Lokuge W., Manalo A., Karunasena W., Frigione M. (2022). Investigation on the Properties of Concrete with Recycled Polyethylene Terephthalate (PET) Granules as Fine Aggregate Replacement. Case Stud. Constr. Mater..

